# Do Patient Characteristics Predict Outcome of Psychodynamic Psychotherapy for Social Anxiety Disorder?

**DOI:** 10.1371/journal.pone.0147165

**Published:** 2016-01-19

**Authors:** Jörg Wiltink, Jürgen Hoyer, Manfred E. Beutel, Christian Ruckes, Stephan Herpertz, Peter Joraschky, Susan Koranyi, Matthias Michal, Björn Nolting, Karin Pöhlmann, Simone Salzer, Bernhard Strauss, Eric Leibing, Falk Leichsenring

**Affiliations:** 1 Department of Psychosomatic Medicine and Psychotherapy, University Medical Center, Johannes Gutenberg University Mainz, Mainz, Germany; 2 Clinical Psychology and Psychotherapy, Technische Universität Dresden, Dresden, Germany; 3 Interdisciplinary Centre for Clinical Trials (IZKS), University Medical Center of the Johannes Gutenberg University Mainz, Mainz, Germany; 4 Clinic of Psychosomatic Medicine and Psychotherapy, LWL-University Clinic Bochum, Ruhr-University Bochum, Bochum, Germany; 5 Clinic for Psychotherapy and Psychosomatic Medicine, Technical University Dresden, Dresden, Germany; 6 Institute of Psychosocial Medicine and Psychotherapy, University Hospital Jena, Jena, Germany; 7 Clinic of Psychosomatics and Psychotherapeutic Medicine, Esslingen, Germany; 8 Clinic of Psychosomatic Medicine and Psychotherapy, University Medicine, Georg-August-University Goettingen, Goettingen, Germany; 9 Clinic of Psychosomatics and Psychotherapy, Justus-Liebig-University Giessen, Giessen, Germany; Leibniz Institute for Neurobiology, GERMANY

## Abstract

**Objectives:**

Little is known about patient characteristics as predictors for outcome in manualized short term psychodynamic psychotherapy (PDT). No study has addressed which patient variables predict outcome of PDT for social anxiety disorder.

**Research Design and Methods:**

In the largest multicenter trial on psychotherapy of social anxiety (SA) to date comparing cognitive therapy, PDT and wait list condition N = 230 patients were assigned to receive PDT, of which N = 166 completed treatment. Treatment outcome was assessed based on diverse parameters such as endstate functioning, remission, response, and drop-out. The relationship between patient characteristics (demographic variables, mental co-morbidity, personality, interpersonal problems) and outcome was analysed using logistic and linear regressions.

**Results:**

Pre-treatment SA predicted up to 39 percent of variance of outcome. Only few additional baseline characteristics predicted better treatment outcome (namely, lower comorbidity and interpersonal problems) with a limited proportion of incremental variance (5.5 to 10 percent), while, e.g., shame, self-esteem or harm avoidance did not.

**Conclusions:**

We argue that the central importance of pre-treatment symptom severity for predicting outcomes should advocate alternative treatment strategies (e.g. longer treatments, combination of psychotherapy and medication) in those who are most disturbed. Given the relatively small amount of variance explained by the other patient characteristics, process variables and patient-therapist interaction should additionally be taken into account in future research.

**Trial Registration:**

Controlled-trials.com/ISRCTN53517394

## Introduction

Patient characteristics and life circumstances are supposed to explain a substantial percentage of variance (about 40%) of the improvement by psychotherapy [1]. Patient characteristics may interact, both with therapist and treatment characteristics, and therefore therapy-patient match may be highly relevant for treatment planning [2, 3].

In their overview on client characteristics, Bohart and Wade classify pre-treatment ‘client contributions’ to outcome into demographic variables, pathology and personal characteristics [4]. Regarding different outcomes of treatment (premature termination, drop-out, response) the authors conclude that demographic variables (age, gender, educational level, socio-economic status, social support and ethnicity) are generally weak or inconsistent predictors [4]. The impact of high symptom severity (as an aspect of pathology) is considered to be two-fold: It may require more sessions and may lead to poorer prognosis [5], but it is also the best predictor of change. The finding that high symptom severity increases the chance to reduce symptoms significantly (change) may seem somewhat counterintuitive. Nevertheless, other outcome variables like response or remission are not necessarily affected by a large reduction of symptoms [4], because of the definition by a reduction of a defined percentage of points on a rating scale (response) or an improvement below a specific cut-off (response). Co-morbidity (e.g. of personality disorders) has been identified as a negative predictor for outcome, but again this finding is not consistent. Finally, results on personal characteristics are yet rather inconsistent, as different measures for diverse constructs have been used across studies and sample sizes are often only small [4, 6].

To date, little is known about patient characteristics as predictors for treatment success specifically in short term psychodynamic psychotherapy. Barber and colleagues recently summarized the results of the few studies relating patient characteristics to outcome of dynamic therapies [2]. Only fragments of the bigger picture of how to predict the later course of therapy could be derived; e.g., outcome was better for minority males and white females in supportive expressive therapy for depression [7], and panic patients with Cluster C personality disorders achieved more improvement by Panic focused Psychodynamic Psychotherapy (PFPP) than patients without personality disorders [8]. For social anxiety disorder, one of the most frequent and debilitating anxiety disorders [9], no study so far has addressed patient characteristics as predictors of psychodynamic psychotherapy.

Patient characteristics may also influence the hypothetical change mechanisms of psychodynamic psychotherapy and therefore affect outcome indirectly. Mechanisms of change of psychodynamic therapy are [2]: fostering insight in unconscious conflicts (insight), increasing use of adaptive defense (defense style), decreasing rigidity in interpersonal perceptions and behaviour (quality of object relations), improving quality of mental representations of relationships and increasing comprehension of own and other mental states (reflective functioning).

In the largest multicenter trial on social anxiety to date comparing cognitive therapy (CBT), psychodynamic therapy (PDT) and wait list control (WL), a wide range of personality, clinical and interpersonal variables has been assessed in addition to severity of illness and demographic data [10]. Psychodynamic treatment was performed in manualized form [11] based on Supportive Expressive Therapy (SET, [12]). The core conflictual relationship theme (CCRT) was identified as the focus of the short-term treatment, including its components wish (W), reaction of others (RO) and reaction of the self (RS).

The main results of the trial were that CBT and PDT were significantly superior to waiting list for both remission and response. CBT was significantly superior to psychodynamic therapy for remission but not for response. Between-group effect sizes for remission and response were small. Secondary outcome measures showed significant differences in favor of CBT for measures of social phobia and interpersonal problems, but not for depression [9]. For both treatments response rates were approximately 70% by the 2-year follow-up. Remission rates were nearly 40% for both treatment conditions. Treatment effects were stable or tended to increase for both treatments over the 24-month follow-up period [13].

Given the broad data base of a sample of patients with social anxiety, for which the diagnosis had been ascertained using highly reliable and valid interviews performed by specifically trained interviewers, our aim was to identify patient characteristics predicting endstate functioning, remission, response and drop-out for PDT in social anxiety disorder using a comprehensive set of patient characteristics.

In an independent analysis of the CBT arm of the multicenter comparative psychotherapy study conducted by the Social Phobia Psychotherapy Research Network (SOPHO-NET; [10, 13, 14]) up to 37 percent of the post-treatment variance (social anxiety) could be explained by all pre-treatment variables combined [15]. Symptom severity (pre-treatment social anxiety), by far, was the strongest predictor, and only small increases of explained variance were achieved by additional variables: comorbid conditions (number of diagnoses) were associated with worse endstate functioning and response. Self-esteem was related to higher endstate functioning and shame was related to *better* response.

In order to identify also the outcome predictors of PDT of social anxiety, we now analysed data of the respective treatment arm of the SOPHO-NET study [10, 13, 14].

Based on several previous studies [4] we expected only small and insignificant effects of *demographic variables* (age, gender, education) on treatment outcome (endstate functioning, remission, response, and drop-out). Patients with higher symptom severity were considered to be more likely to improve during treatment in terms of their scoring on a symptom scale, but to be less likely to reach the remission criterion (symptom severity below a defined score on a symptom scale). Therefore, regarding the initial severity of *social anxiety* we expected participants with higher symptom severity to have *higher* endstate functioning, to respond *more* frequently and to remit and stay in treatment (drop-out) *less* frequently. We further expected that *mental co-morbidity* reflected by the number of co-morbid mental disorders and the intensity of pre-treatment symptoms of *depression* would have a negative impact on outcome (endstate functioning, remission, response, and drop-out). This relationship has previously been shown for CBT in social anxiety [6]. Regarding *personal characteristics* of the participants, we assessed pre-treatment shame, self-esteem and personality variables (based on Cloninger’s concept of personality [16], which includes harm avoidance, novelty seeking and reward dependence) in our trial. As shame is the central emotion in social anxiety [17], Leichsenring et al. put particular emphasis on the focus of shame in their treatment manual on PDT of social anxiety disorder [11]. In terms of the core conflictual relationship theme (CCRT), shame can be regarded as a response of the self (RS) towards devaluating objects (RO). It may play an important role in maintaining a vicious circle of social anxiety and avoidance. Low *self-esteem* also plays a major role in social anxiety disorder [18] and it is supposed to influence both the anticipated reaction of others and the response of self (RO and RS component of the CCRT). Therefore, we expected that patients who are initially overwhelmed by shame and those with low self-esteem would show a poorer treatment outcome.

While evidence supporting personality theories (e.g. [16]) is growing, *personality factors* have not been regularly assessed as predictor variables in psychotherapy studies. As there is some evidence that high ‘harm avoidance’—characterized by excessive worrying, pessimism, shyness, being fearful, doubtful, and easily fatigued—is related to poor treatment outcome in individual, group CBT and usual treatment of social anxiety we expected high ‘harm avoidance’ also to have a negative effect on treatment outcome in PDT [19].

*Interpersonal problems* such as hostility, which have been frequently identified in social anxiety (e.g. [20]), may also negatively affect treatment outcome as has been shown by Horowitz et al. [21]. High anxiety and avoidance in close relationships as attachment representations, may interfere with the implementation of specific interventions in a manualized treatment approach and are therefore expected to be associated with lower treatment success (endstate functioning, response, remission, and drop-out). As a recent meta-analysis indicates, *attachment characteristics* of the patients might be additional predictors of treatment outcome. Levy et al. showed that attachment anxiety is associated with less positive outcome in a wide range of psychotherapeutic treatments [22].

## Material and Methods

### Study Design and Implementation

Date range from patients’ recruitment and follow-up was from April 11, 2007, to May 15, 2012. Patients were recruited by outpatient clinics at the universities of Bochum, Dresden, Goettingen, Jena, and Mainz. At each of the five centers one clinic performed psychodynamic therapy and another performed CBT, investigator allegiance effect was further controlled by including experts in both CBT and PDT as local investigators at each center. The study protocol was approved by the responsible ethics committee of the Georg-August University Göttingen, 14/5/06 and conducted in accordance with the guidelines for good clinical practice (www.ema.europa.eu/ema/). The study was monitored by the Coordination Center for Clinical Trials at Heidelberg (http://www.klinikum.uni-heidelberg.de/KKS-Heidelberg.2411.0.html), which is independent of the participating research centers. Providing written informed consent was required for inclusion.

Detailed information on the study is provided elsewhere [10, 13, 14].

### Treatment

A manual guided-form of psychodynamic therapy was specifically developed for this trial. It was based on Luborsky’s model of psychodynamic therapy (Supportive Expressive Therapy, [12]) which was adapted to treat social anxiety disorder. This model encompasses both supportive and expressive interventions that are assumed to lead to therapeutic change [11, 12]. Establishing a secure helping alliance is one of the model’s most important supportive treatment elements. Expressive interventions relate the symptoms of social anxiety disorder to the patient’s underlying core conflictual relationship theme (CCRT)—explained in the introduction—in order to reduce the symptoms of social anxiety disorder [11, 12]. The response from the self represents the symptoms of social anxiety disorder. A therapist could link the components of the CCRT by an expressive intervention [11]: “As we have seen, you are not only afraid of exposing yourself (response from the self), but you sometimes wish to be at the center of attention and to be affirmed by others (wish). However, you are afraid that they will humiliate you (response from others).” The CCRT is worked through in present and past relationships as well as in the relationship to the therapist. The treatment procedures are described in detail in a manual [11].

Up to 25 individual (50-minute) treatment sessions were applied. In addition, up to 5 preparatory sessions were conducted which are required in the German health care system to cover diagnostic and administrative issues. The mean number of sessions completed was 25.7 (SD = 9.61). The mean duration of treatment was 37.4 weeks (SD = 18.03) for psychodynamic therapy.

### Study Subjects

The following inclusion criteria were applied: Age range of 18–70 years, diagnosis of social anxiety disorder according to Structured Clinical Interview (SCID I) for DSM-IV [23], Liebowitz-Social-Anxiety-Scale >30 [24], and a primary diagnosis of social anxiety disorder according to the rating of the Anxiety-Disorders-Interview-Schedule scale [25]. In order to recruit a clinically representative sample, all co-morbid mental disorders less severe than social anxiety disorder (according to the Anxiety-Disorders-Interview-Schedule scale rating) were permitted except for the conditions listed among the following exclusion criteria: psychotic and acute substance-related disorders; cluster A and B personality disorders; prominent risk of self-harm; organic mental disorders; severe medical conditions; subjects with concurrent psychotherapeutic or psychopharmacological treatments were also excluded. Providing written informed consent was required for inclusion.

For the analyses presented in this paper only data of the PDT treatment arm of the study were used. The characteristics of the study completers and those who dropped out are listed in Table 1.

**Table 1:** Sample characteristics (N = 197–230) ^1)^.

	Completers (N = 147–167)	Drop-outs (N = 50–63)
Demographic data		
Age	34.46 (12.1)	35.73 (12.65)
Sex, female	51.5%	55.6%
Education, at least high school certificate	69.5%	44.4%
Partnership	43.1%	30.2%
Social Anxiety (LSAS pre)	71.35 (21.06)	79.46 (22.85)
Co-morbidity		
Number of mental diagnoses	0.90 (0.92)	1.32 (1.08)
Depression (BDI)	13.58 (9.16)	15.21 (10.22)
Personality		
Shame (Tosca)	46.58 (9.24)	44.67 (13.20)
Harm avoidance (TPQ)	23.85 (5.00)	23.79 (5.19)
Novelty seeking (TPQ)	12.90 (4.73)	13.28 (5.22)
Reward dependence (TPQ)	16.33 (4.37)	16.00 (4.32)
Self-esteem (FSKN)	36.93 (9.56)	38.57 (11.05)
Interpersonal problems		
Love (IIP)	-.26 (.62)	-.40 (.58)
Dominance (IIP)	-.90 (.58)	-.92 (.71)
Anxiety in close relationships (ECR-Anx)	3.55 (1.19)	3.66 (1.41)
Avoidance in close relationships (ECR-Avo)	3.03 (1.10)	2.84 (1.07)

^1)^ including patients previously on waitlist assigned to receive PDT

### Therapists

All study therapists held degrees as clinical psychologists or physicians. They had completed their psychotherapeutic training or were in advanced psychotherapeutic training. Fifty-three psychodynamic therapists conducted psychodynamic therapy (30 female). Mean age of therapists was 39.44 (SD = 8.06). The average clinical experience was 8.0 (SD = 9.0) years.

Before inclusion in the trial, therapists were specifically trained in the manualized treatment approach by authors of the treatment manual (MB, FL). In order to be included in the trial, therapists were required to treat two pilot cases in accordance with the respective manual. In order to maintain treatment fidelity during the trial, therapists received regular site-level and cross-site supervision. At each center, supervision of therapists was performed as a group supervision of 90 minutes conducted by supervisors specifically trained by the authors of the treatment manual (MB, FL). During the first six months of the trial, supervision was performed fortnightly and monthly thereafter. All treatment sessions were videotaped. Video tapes were used for both supervision and the assessment of treatment integrity.

### Patient Flow

Patients were allocated to psychodynamic therapy, cognitive therapy or waiting list in a ratio of 3:3:1. This ratio with a relatively low number of participants initially randomized to wait list ensured a sufficient power for comparisons of treatments with wait list. For details of randomization see Leichsenring et al. [13, 14].

**Fig 1:**
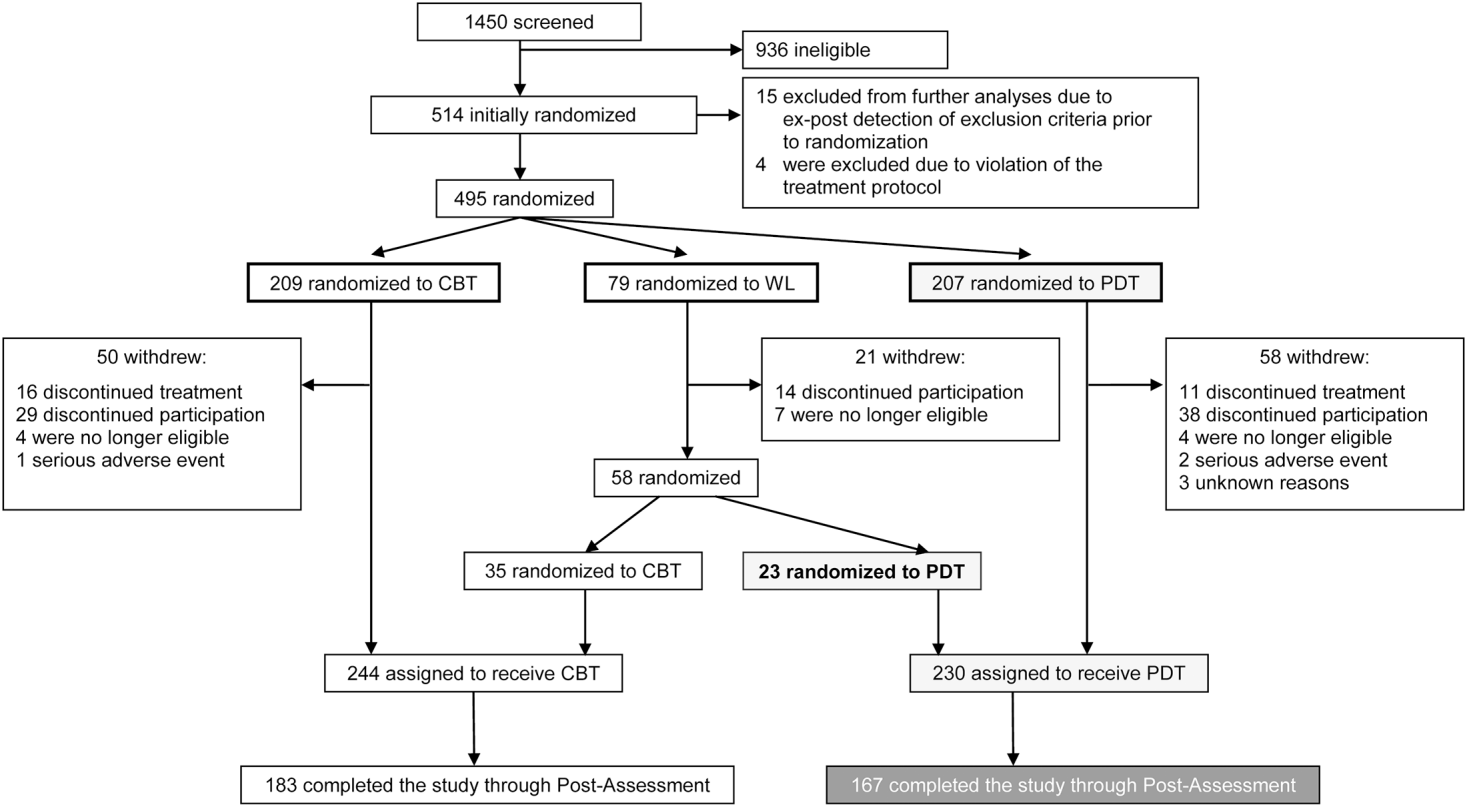
Patient flow.

Fig 1 summarizes the patient flow.

### Assessment

Assessments were conducted at baseline, at weeks 8 and 15 of treatment and post-treatment. Diagnoses were made by use of SCID (I, II) [23]. Using the Anxiety-Disorders-Interview-Schedule rating scale, the primary (i.e., most severe) mental disorder was assessed [25]. Twenty-three specifically trained and independent assessors (clinical psychologists) masked to the treatment conditions conducted the interviews. All interviews were videotaped in order to assess inter-rater reliability. Reliability for the Liebowitz-Social-Anxiety-Scale was assessed by comparing the individual results of 23 diagnosticians to an expert’s rating of three videotaped interviews. High inter-rater reliability was observed for the Liebowitz-Social-Anxiety-Scale (total score; Kendall’s W = 0.98; df = 2; p<0.001).

#### Outcomes (dependent Variables)

The primary outcomes response and remission were based on the Liebowitz-Social-Anxiety-Scale (LSAS) [10]. Following recommendations by [26], *remission* was defined by a Liebowitz-Social-Anxiety-Scale score ≤30 [24, 26]. *Response* was defined by a 31% reduction (or more) in the Liebowitz-Social-Anxiety-Scale which is comparable to Clinical Global Impression Improvement scale score ≤2 usually used to define response [27].

*Endstate functioning* of social anxiety was defined as the post treatment LSAS score. All patients who stopped treatment or assessment (treatment or study withdrawals) were defined as *drop-outs*. While drop-out is commonly associated with poor outcome, the course of symptoms in drop-outs is usually unclear. We chose this outcome not to mix unclear with documented poor outcome.

#### Potential Predictors (independent Variables)

In addition to the initial social anxiety (LSAS) as an autoregressor we used the following independent variables:

1. Demographic variables: The following demographic characteristics were used as potential predictors: age, gender and education. The different educational levels were dichotomized into 1 ‘finished high school’ and 0 ‘did not finish high school’.
2. Mental co-morbidity: Mental co-morbidity was measured by the number of mental diagnoses including personality disorders assessed by Structured Clinical Interview (SCID I and II) for DSM-IV [23]. Depressive symptoms were measured by the Beck Depression Inventory (BDI, [28]). The BDI is a reliable self-rating instrument with a sufficient internal consistency (Cronbach’s alpha 0.84 and a retest-reliability of r = 0.75. It has been proven to discriminate validly between different levels of depression [29].
3. Personality: We used the ‘shame’ subscale of the German translation of the Test Of Self-Conscious Affects (TOSCA, [30]). This subscale consists of 15 items presenting social scenarios to be rated regarding possible reactions (shame, guilt) on a 5-point Likert scale (1 ‘not likely’ to 5 ‘very likely’). The scale achieved good internal consistency (Cronbach’s alpha 0.90 in a clinical sample, [30]). Personality dimensions were assessed with the German version of the Tridimensional Personality Questionnaire (TPQ, [16, 31]). The internal consistencies of the scales Novelty Seeking (NS), Harm Avoidance (HA) and Reward Dependence (RD) were high and factor analysis of the TPQ supported Cloninger’s personality theory [31]. Self-esteem (FSSW) was assessed with a subscale of the Frankfurt Self Concept Scales (FSKS, [32]). The scale consists of 10 items to be answered using a 6-point Likert scale (1 “applies completely” to 6 “does not apply at all”).
4. Interpersonal problems and Attachment: The German version of the Inventory of Interpersonal Problems (IIP-D) measures self-rated difficulties with other people. Sixty-four items are subsumed under eight scales (eight items each related to the interpersonal circumplex): overly domineering, vindictive, cold, socially avoidant, nonassertive, exploitable, nurturant, and intrusive [20, 33, 34]. In order to reduce the number of variables, only the two major dimensions of the interpersonal circle were analysed [35]. Behaviour incorporated in the horizontal axis (commonly referred to *love* in IIP literature) ranges from excessively sacrificing one’s own needs in favour of others to lacking care for others and feeling interpersonally detached. On the other hand, the vertical axis represents problems with *dominance* (i.e., status and control) and extends from being overly controlling to being insufficiently assertive.

Romantic attachment representations were assessed with the two subscales “anxiety” and “avoidance” in close relationships of the German version of the ‘Experiences in Close Relationships–Revised’ questionnaire (ECR-R, [36]).

Table 2 gives an overview regarding potential predictors.

**Table 2:** Potential Predictors.

Predictor	Assessment
Social Anxiety	Liebowitz-Social-Anxiety-Scale (LSAS, [24])
Demographic data	Self-report
Age	
Sex, female	
Education, at least high school certificate	
Co-morbidity	
Number of mental diagnoses	Structured Clinical Interview (SCID I and II) for DSM-IV [23]
Depression (BDI)	Beck Depression Inventory (BDI, [28])
Personality	
Shame (Tosca)	Test Of Self-Conscious Affects (TOSCA, [30])
Harm avoidance (TPQ)	Tridimensional Personality Questionnaire (TPQ, [16, 31])
Novelty seeking (TPQ)	
Reward dependence (TPQ)	
Self-esteem (FSKN)	Frankfurt Self Concept Scales (FSKS, [32])
Interpersonal problems	
Love (IIP)	Inventory of Interpersonal Problems (IIP-D, [33, 35])
Dominance (IIP)	
Anxiety in close relationships (ECR-Anx)	Experiences in Close Relationships–Revised’ questionnaire (ECR-R, [36])
Avoidance in close relationships (ECR-Avo)	

### Statistical Analyses

Statistical analyses were computed using IBM SPSS Statistics 20 (IBM, Chicago, IL).

The analyses were performed according to the procedure described by Hoyer et al., who investigated the predictors of the CBT arm of the same study (SOPHO-NET) [15].

Bivariate correlations between patient characteristics and outcome data were determined using Pearson´s r (LSAS pre, endstate functioning) and Spearman´s Rho coefficients (response, remission, drop-out). Reported p-values (correlations, see Table 3) correspond to 2-tailed tests and were considered significant at the .00067 level (Bonferroni adjustment; 0.05/ 75 tests).

**Table 3:** Correlations between patient characteristics, pre-treatment social anxiety and different outcomes (endstate functioning, response, remission, drop-out).

		LSAS pre (N = 147–166) ^1)^	Endstate (N = 147–166) ^1)^	Response (N = 147–166) ^2)^	Remission (N = 147–166) ^2)^	Drop-out (N = 197–230) ^2)^
1	Symptom severity					
	Social Anxiety (LSAS pre)		.645*	-.085	-.433*	.162
2	Demographic data					
	Age	-.116	-.148	.093	.121	.043
	Sex	-.060	-.013	.086	-.002	.034
	Education	-.064	.058	-.096	-.137	-.177
3	Co-morbidity					
	Number of mental diagnoses	.350*	.298*	-.159	-.184	.177
	Depression (BDI)	.397*	.361*	-.104	-.207	.063
4	Personality					
	Shame (Tosca)	.388*	.417*	-.166	-.284*	-.086
	Harm avoidance (TPI)	.477*	.408*	-.109	-.310*	-.019
	Novelty seeking (TPI)	-.121	-.148	.060	.126	.029
	Reward dependence (TPI)	-.115	-.064	.045	.039	-.048
	Self-esteem (FSKN)	-.427*	-.379*	.110	.304*	.060
5	Interpersonal problems					
	Love (IIP)	-.290*	-.118	-.114	.083	-.121
	Dominance (IIP)	-.355*	-.279*	.162	.191	-.025
	Anxiety in close relationships (ECR-Anx)	-.330*	.320*	-.090	-.224*	.036
	Avoidance in close relationships (ECR-Avo)	.386*	.232*	.097	-.176	-.066

^1)^ Pearson correlations;

^2)^ Spearman Rho correlations;

* p < .00067 (after Bonferroni adjustment)

To analyse the relationship between endstate functioning and potential patient characteristics (demographic variables, mental co-morbidity, personality, interpersonal problems) we used linear regression models with endstate functioning (LSAS post) as the dependent variable and patient characteristics as independent variables (predictors). We entered variables blockwise as defined above. Model 1 describes the predictive value of pre-treatment social anxiety (LSAS pre), models 2 to 5 included the above mentioned additional independent variables.

To determine the relationship between remission, response, drop-out and potential patient characteristics (demographic variables, mental co-morbidity, personality, interpersonal problems), we used logistic regression models with remission, response and drop-out as the dependent variable and patient characteristics as independent variables. Equivalent to the linear regression, we entered variables blockwise. Goodness of fit was tested by Hosmer-Lemeshow-Test for each block.

Shame and self-esteem were integrated into the block of personality variables, given the typically high retest-reliability of these variables [30, 32].

We used an inclusion criterion of p<0.05, and an exclusion criterion of p>0.10 for the regression analyses. Reported p-values (regressions) correspond to 2-tailed tests and were considered significant at the .05 level.

## Results

### Bivariate Relations between Patient Characteristics and Outcome

Table 3 shows the bivariate correlations between predictors and outcome. In order to increase readability, predictors were organized according to the blocks used in the regression analyses: symptom severity, demographic data, co-morbidity, personality, interpersonal problems and pre-treatment social anxiety resp. different outcomes (endstate functioning, response, remission, and drop-out).

Endstate functioning and remission, but not response and dropout were related to symptom severity (pre-treatment social anxiety). As expected, demographic characteristics (age, education and sex) were unrelated to any of the outcome indicators. The number of mental diagnoses and depression as indicators of comorbidities were related with higher pre-treatment and also higher post-treatment social anxiety (endstate functioning). Among the personality characteristics, shame, harm avoidance and self-esteem were consistently related to social anxiety (pre-treatment and endstate functioning) and remission, but not to response and drop-out. Novelty-seeking and reward dependence were unrelated to any outcome indicator. The ‘Dominance’-scale (IIP) was significantly correlated with endstate functioning. Both anxiety and avoidance in close relationships (ECR) were substantially correlated with endstate functioning, while anxiety in close relationships (ECR) was only related to remission. There was no bivariate relation between any of the investigated interpersonal problems and response and drop-out.

### Predicting Endstate Functioning

In our linear regression model with a block wise entering of variables higher endstate functioning was predicted by block 1 (higher pre-treatment social anxiety), explaining a total of 39 percent of variance of end-state social anxiety. Block 3 (co-morbidity) added substantial 5.5% of variance (see Table 4). Within this block, depression was positively correlated with higher end state social anxiety (ß = 0.199, p = .032), while the number of mental diagnoses was not (ß = .090, p = .212). Block 2 (demographic variables), block 4 (personality) and block 5 (interpersonal problems) were not significantly related to end state social anxiety.

**Table 4:** Patient characteristics predicting endstate functioning (LSAS post); N = 136.

Model/ Block	Predictor variables ^1)^	ß ^2)^	p(ß) ^2)^	R^2^	Adj. R^2^	R^2^-Change	F-Change	p (F-Change)
1	LSAS pre	.541	< .001	.391	.387	.391	86.059	< .001
2	Age	-.140	.061	.404	.386	.013	.969	.410
	Sex	.040	.577					
	Education	.067	.342					
3	Number of mental diagnoses	.090	.212	.460	.434	.055	6.597	.002
	Depression (BDI)	.199	.032					
4	Shame (Tosca)	.151	.087	.477	.430	.017	.809	.546
	Harm avoidance (TPQ)	-.031	.758					
	Novelty seeking (TPQ)	-.058	.416					
	Reward dependence (TPQ)	-.176	.048					
	Self-esteem (FSKN)	.012	.905					
5	Love (IIP)	.237	.010	.516	.456	.039	2.443	.050
	Dominance (IIP)	.100	.199					
	Anxiety in close relationships (ECR-Anx)	.105	.200					
	Avoidance in close relationships (ECR-Avo)	-.107	.212					

^1)^ Linear regression model

^2)^ Corresponding to the final model

### Predicting Response

The logistic regression model (block wise entering of variables) suggests that there was no association between pre-treatment social anxiety and treatment response. In this model, we found a significant relation between block 3 (co-morbidity) explaining 8.1 percent of variance and block 5 (interpersonal problems) explaining 10 percent of variance to treatment response (see Table 4). Only the number of mental diagnoses (comorbidity) was negatively related to response (OR = .594 [.371/ .952], p = .030). The variable love (IIP) of block 5 (interpersonal problems) was negatively related to response (OR = .311 [.123/ .791], p = .014) as well, however the Hosmer-Lemeshow-Test (p = .008) for the model including block 5 indicated a poor goodness of fit. Block 2 (demographic variables) and the five variables of block 4 (personality) did not reach significance in a regression analysis as predictors of response. See Table 5.

**Table 5:** Patient characteristics predicting response; N = 136.

Model/ Block	Predictor variables ^1)^	OR ^2)^	95%CI ^2)^	p(OR) ^2)^	R^2^ ^3)^	X^2^-Change	p (X^2^-Change)
1	LSAS pre	1.001	.977/ 1.026	.928	.003	.306	.580 ^4)^
2	Age	1.027	.990/ 1.066	.153	.014	1.142	.767 ^5)^
	Sex	1.589	.671/ 3.763	.292			
	Education	.540	.206/ 1.417	.211			
3	Number of mental diagnoses	.594	.371/ .952	.030	.081	7.001	.030 ^6)^
	Depression (BDI)	.955	.900/ 1.013	.126			
4	Shame (Tosca)	.948	.895/ 1.004	.070	.123	4.607	.466 ^7)^
	Harm avoidance (TPQ)	1.053	.937/ 1.184	.381			
	Novelty seeking (TPQ)	1.020	.932/ 1.118	.663			
	Reward dependence (TPQ)	1.107	.979/ 1.251	.104			
	Self-esteem (FSKN)	.975	.916/ 1.038	.422			
5	Love (IIP)	.311	.123/ .791	.014	.223	11.541	.021 ^8)^
	Dominance (IIP)	1.080	.449/ 2.598	.864			
	Anxiety in close relationships (ECR-Anx)	.753	.496/ 1.145	.184			
	Avoidance in close relationships (ECR-Avo)	1.529	.935/ 2.502	.091			

^1)^ Logistic regression model

^2)^ Corresponding to the final model.

^3)^ Nagelkerkes R^2^

Hosmer-Lemeshow-Test for goodness of fit:

^4)^ X^2^(8) = 3.5, p = .90;

^5)^ X^2^(8) = 3.9, p = .87;

^6)^ X^2^(8) = 12.7, p = .12;

^7)^ X^2^(8) = 5.7, p = .68;

^8)^ X^2^(8) = 20.6, p = .008

### Predicting Remission

Remission was only predicted by block 1 (pre-treatment social anxiety). None of the other blocks of variables added a substantial amount of variance (see Table 6). Pre-treatment social anxiety predicted a total of 27.2 percent of variance.

**Table 6:** Patient characteristics predicting remission; N = 136.

Model/ Block	Predictor variables ^1)^	OR ^2)^	95%CI ^2)^	p(OR) ^2)^	R^2^ ^3)^	X^2^-Change	P (X^2^-Change)
1	LSAS pre	.931	.898/ .966	< .001	.272	29.372	< .001 ^4)^
2	Age	1.035	.993/ 1.079	.106	.326	6.823	.078 ^5)^
	Sex	.923	.320/ 2.662	.882			
	Education	.264	.084/ .828	.022			
3	Number of mental diagnoses	.969	.548/ 1.713	.914	.334	.765	.682 ^6)^
	Depression (BDI)	1.054	.976/ 1.137	.181			
4	Shame (Tosca)	.957	.894/ 1.023	.198	.407	9.612	.087 ^7)^
	Harm avoidance (TPQ)	1.013	.876/ 1.170	.863			
	Novelty seeking (TPQ)	1.103	.985/ 1.235	.090			
	Reward dependence (TPQ)	1.083	.945/ 1.241	.252			
	Self-esteem (FSKN)	1.080	1.004/ 1.161	.039			
5	Love (IIP)	.303	.102/ .895	.031	.447	5.632	.228 ^8)^
	Dominance (IIP)	.469	.172/ 1.281	.140			
	Anxiety in close relationships (ECR-Anx)	1.002	.620/ 1.619	.993			
	Avoidance in close relationships (ECR-Avo)	.916	.511/ 1.642	.769			

^1)^ Logistic regression model

^2)^ Corresponding to the final model.

^3)^ Nagelkerkes R^2^

Hosmer-Lemeshow-Test for goodness of fit:

^4)^ X^2^(8) = 2.7, p = .95;

^5)^ X^2^(8) = 6.8, p = .56;

^6)^ X^2^(8) = 7.7, p = .47;

^7)^ X^2^(8) = 9.2, p = .32;

^8)^ X^2^(8) = 16.6, p = .034

### Predicting Drop-out

Regarding all kinds of dropouts, our logistic regression model suggests a significant relation between block 1 (higher pre-treatment social anxiety) and drop-out rates. (see Table 7). 4.8 percent of variance was predicted by pre-treatment social anxiety.

**Table 7:** Patient characteristics predicting drop-out; N = 180.

Model/ Block	Predictor variables ^1)^	OR ^2)^	95%CI ^2)^	p(OR) ^2)^	R^2^ ^3)^	X^2^-Change	p (X^2^-Change)
1	LSAS pre	1.023	.999/ 1.048	.064	.048	5.939	.015 ^4)^
2	Age	1.009	.976/ 1.044	.591	.078	3.661	.300 ^5)^
	Sex	1.116	.478/ 2.606	.800			
	Education	.474	.247/ 1.333	.196			
3	Number of mental diagnoses	1.280	.822/ 1.994	.275	.090	1.548	.461 ^6)^
	Depression (BDI)	1.057	.997/ 1.122	.064			
4	Shame (Tosca)	.993	.946/ 1.044	.791	.154	8.434	.134 ^7)^
	Harm avoidance (TPQ)	.936	.842/ 1.042	.226			
	Novelty seeking (TPQ)	1.040	.954/ 1.133	.373			
	Reward dependence (TPQ)	1.029	.917/ 1.155	.623			
	Self-esteem (FSKN)	1.070	1.007/ 1.137	.029			
5	Love (IIP)	.530	.226/ 1.247	.146	.206	7.190	.126 ^8)^
	Dominance (IIP)	.621	.276/ 1.394	.248			
	Anxiety in close relationships (ECR-Anx)	1.462	.994/ 2.150	.054			
	Avoidance in close relationships (ECR-Avo)	.636	.389/ 1.041	.072			

^1)^ Logistic regression model

^2)^ Corresponding to the final model.

^3)^ Nagelkerkes R^2^

Hosmer-Lemeshow-Test for goodness of fit:

^4)^ X^2^(8) = 5.6, p = .70;

^5)^ X^2^(8) = 9.7, p = .29;

^6)^ X^2^(8) = 14.7, p = .07;

^7)^ X^2^(8) = 7.8, p = .45;

^8)^ X^2^(8) = 8.6, p = .38

## Discussion and Conclusions

In our large multicenter trial on social anxiety we could assess and analyse a broad range of potential predictors of treatment outcome [10], including psychopathology, personality, and interpersonal variables, most of which had never been investigated in PDT outcome studies before. Our aim was to identify patient characteristics predicting the central defining parameters of outcome (endstate functioning, remission, response and drop-out) of PDT for social anxiety disorder. Bivariate correlations indicate that demographic variables, as well as mental co-morbidity, personality and interpersonal problems (without correction for potential confounders) were significantly related to outcome (endstate functioning and remission). However, after controlling for pre-treatment social anxiety our regression models suggested that only few baseline characteristics still significantly predict treatment outcome on a multivariate level. Clearly, high pre-treatment social anxiety was found to be the strongest predictor of higher endstate functioning, lower remission and–less pronounced–drop-out from treatment. The predictive value of other patient characteristics beyond baseline social anxiety remained only small, however comparable to findings on CBT of social anxiety [6, 15].

In addition to higher baseline social anxiety, depression was a predictor for a higher post-treatment social anxiety (endstate functioning). While pre-treatment social anxiety did not significantly predict response, more mental diagnoses—indicating more comorbidity—and higher scores on the IIP-dimension Love (indicating a tendency of excessively sacrificing one’s own needs) predicted less response in our multivariate model. The only patient characteristic related to remission and drop-out was higher pre-treatment social anxiety.

Similar to Hoyer et al. [15] we also did not find relations between demographic variables and different outcomes on a bivariate level. Also with regard to comorbidity, our correlations between the number of mental diagnoses and outcomes were similar [15]. However, the number of mental diagnoses did not significantly correlate with response and remission due to a more conservative approach (Bonferroni adjustment). In contrast to Hoyer et al. [15], depression was significantly related to worse endstate functioning. Again, on a bivariate level, we found the same relations between personality and different outcomes. Interestingly, in our analyses higher pre-treatment shame–as a central emotion in social anxiety–was substantially related to worse outcome (endstate, remission) bivariately, but in the regression models shame explained only an insignificant portion of variance in addition to pre-treatment social anxiety.

The largest differences to the CBT arm were obtained for interpersonal problems: In the PDT arm Love (IIP) was unrelated to outcomes in bivariate analyses, while attachment (higher anxiety and higher avoidance in close relationships) was related to worse endstate functioning and remission.

Summarizing, like Hoyer et al. [15] did for the CBT data, we found pre-treatment social anxiety to be by far the strongest predictor on a multivariate level, with comorbidity explaining limited additional variance. Thus, overall, the incremental variance explained by patient characteristics beyond pre-treatment social anxiety was small. Most of our initial bivariate relations to outcome variables were lost by multivariate analyses. Remarkably, this applies also to interpersonal problems when related to endstate functioning and drop-out multivariately. These findings, thus, contradict the expectations that some manifestations of interpersonal problems may–at least mediated by a negative impact on the therapeutic alliance [37]–complicate the therapeutic process and (negatively) interfere with success [21]. Indeed, interpersonal problems are significantly related to social anxiety severity at treatment start (see Table 2), but the two should not be confused, and–except the mentioned predictive value of the dimension Love for response–only social anxiety severity itself is truly relevant for the course of treatment and outcome.

The strong effect of pre-treatment social anxiety on outcome implies that patients starting treatment with higher social anxiety are still those most affected by symptoms after treatment and those who are less likely to remit. These results might suggest that alternative treatment strategies should be preferred for those patients with a higher initial symptom severity. One strategy could be a longer treatment duration (extending the maximum of 25 plus up to 5 preparatory sessions that were allowed in the study) for achieving a better outcome. It is an intriguing question for future studies on the efficacy of PDT in social anxiety disorder then, whether a symptom severity adapted treatment duration could decrease the large effect of pre-treatment social anxiety on outcome. As suggested by recent guidelines on the treatment of anxiety disorders [38] or meta-analyses (e.g. [39]), a combination of psychotherapy and medication treatment may also be helpful for those with worse outcome or higher initial symptom severity. Another option is to switch the psychotherapeutic approach when the first treatment has not proven to be sufficiently effective [38] as recently demonstrated by Gloster et al. [40].

The strengths of our study include the relatively large sample size and the broad assessment of potential predictors. Limitations pertain to the focus on pre-treatment characteristics that do not take into account possible interactions between patient and therapist or process variables (e.g. adherence, competence to treatment protocol). The additional predictive value of pre-treatment characteristics varies substantially between the dependent variables investigated (endstate functioning, response, remission, and drop-out), which is in part attributable to simple statistical reasons: While the endstate functioning criterion is obtained from scores at a discrete point of time (post-treatment), remission and response reflect change of scores over time, indicating that different hypotheses are investigated by the regression models. For example, remission as outcome (defined by a Liebowitz-Social-Anxiety-Scale score ≤30) is only predicted by pre-treatment social anxiety (27% of variance explained) with no further predictors. The reason for this result might be that patients with lower pre-treatment (e.g. LSAS score = 35) social anxiety have an extremely good chance to fall below a score of 30, whereas it is much harder for those with higher scores. In contrast, most significant predictors—not including pre-treatment social anxiety—were found for response (defined by at least 31% reduction in LSAS) reflecting that response is possible in the group of patients with highest social anxiety and also in the group of patients with moderate social anxiety. Therefore, it is more likely that instead of pre-treatment social anxiety criterion variance is captured by other patient characteristics.

Future research including patient characteristics should focus on differential indications for different treatment options [2]. Given the overall small amount of variance explained by patient characteristics in the present study, the role of process variables and of the patient-therapist interaction should be taken into account to better mirror the full complexity of the psychodynamic treatment and the dynamic interactions between variables predicting its course and outcome.
